# Objectifying persistent subjective cognitive impairment following COVID-19 infection: cross-sectional data from an outpatient memory-clinic in Germany

**DOI:** 10.1007/s00406-025-01978-1

**Published:** 2025-02-28

**Authors:** Luca Tarantini, Corina Möller, Victoria Schiestl, Sabrina Sordon, Michael Noll-Hussong, Miriam Wittemann, Nicole Menzie, Matthias Riemenschneider

**Affiliations:** 1 Department of Psychiatry and Psychotherapy, Saarland University Medical Center, Kirrberger Straße, Building 90, 66421 Homburg, Germany; 2 Psychosomatic Medicine and Psychotherapy, Day-Hospital Westend, Munich, Germany

**Keywords:** COVID-19, Post-COVID, Cognitive impairment, Cognitive complaints, Depression, APOE-ε4

## Abstract

**Objective:**

Subjective cognitive impairment is frequently reported by patients experiencing Post-COVID symptoms. This study aims to assess objective impairment in attention, memory, and executive functions among these patients. Further, we investigated potential determinants of objective cognitive impairment.

**Methods:**

In this cross-sectional study, standardized neuropsychological testing (Vienna Testing System), assessment of cognitive symptom aggravation, psychiatric anamnesis, and psychometrics (BDI-II, Fatigue Severity Scale) were conducted in 229 patients who voluntarily presented to our outpatient memory-clinic due to subjective cognitive impairment following COVID-19. Blood-samples were collected to assess peripheral immune markers (IL-6, CRP) and APOE-ε4 genotype.

**Results:**

Objective cognitive impairment in at least one domain was present in 39% of the patients and 47% showed symptoms of moderate or severe depression. The *APOE*-ε4 allele was present in 32% of the patients. Higher rates of depressive symptoms (OR = 1.41, 95%-CI = 1.02–1.95) and higher burden of the *APOE*-ε4 allele (OR = 3.29, 95%-CI = 1.51–7.40) predicted objective cognitive impairment, regardless of age, sex, years of formal education, time since infection, and medication for diabetes or hypertension. Fatigue severity, acute COVID-19 severity or inflammation markers had no impact.

**Conclusions:**

In our study, subjective cognitive impairment following COVID-19 was more likely associated with high rates of depression rather than relatively low rates of objective cognitive performance. Thus, the study emphasizes the necessity for extensive neuropsychological testing and evaluation of depression when examining Post-COVID patients in clinical practice. Further, the link between objective cognitive impairment, depression and APOE-ε4 does not appear to be specific to Post-COVID symptoms. Therefore, depression- and APOE-ε4-mediated neurodegenerative pathomechanisms might be a promising therapeutical target.

## Introduction

To date, more than 776 million confirmed cases of COVID-19 have been globally reported by the World Health Organization (WHO, WHO Coronavirus (COVID-19) Dashboard | WHO Coronavirus (COVID-19) Dashboard with Vaccination Data). Approximately 10–30% of non-hospitalized and up to 70% of hospitalized patients report persistent health conditions after recovering from the acute phase [1], which is referred to as a Post-COVID or Long COVID condition.

Post-COVID is considered as a multisystemic condition with the presence of a variety of potential symptoms across multiple organ systems [1, 2]. Research has shown that the neuropsychiatric sequelae of COVID-19 encompasses depression, anxiety, symptoms of post-traumatic stress disorder, sleep disorders, cognitive impairment, and fatigue [3], as well as an increased risk for cognitive impairment, seizures, dementia and psychosis for at least two years after the acute infection [4]. In order to identify possible treatment options, determinants of cognitive impairment Post-COVID should be investigated. However, in many studies so far, cognitive performance was measured by means of subjective reports or simple screening instruments [5], whereas extensive neuropsychological assessment is necessary [6].

Recent research discusses various mechanisms of action underlying the effects of COVID-19 on the central nervous system (CNS), such as a sustained dysregulation of the immune system [6, 7]. It has been shown that pro-inflammatory cytokines such as interleukin 6 (IL-6) are persistently elevated among Post-COVID populations [8]. An alternative explanation is that cognitive impairment following COVID-19 might be mediated by depressive symptomology. Approximately 30–40% of patients have been found to suffer from clinically significant depression one, three and six months following COVID-19 [9, 10]. Prior research indicates that patients with depression demonstrate cognitive deficits even after remission [11]. Therefore, Post-COVID depression might be partly responsible for cognitive impairment [6]. Moreover, it has been suggested that presence of the ε4 allele of the apolipoprotein E (APOE-ε4) might lead to cognitive impairment in Post-COVID patients, as it contributes to multiple detrimental mechanisms and pathways related to CNS functioning [12]. Research findings on the latter is mixed, such that one previous study links *APOE*-ε4 to impaired cognition in Post-COVID patients [13], whereas two others do not [14, 15]. This might be due to varying sample sizes and screening procedures to evaluate cognitive performance.

This study focused on a specific subset of patients who voluntarily sought treatment at our outpatient memory-clinic because of novel or exacerbated subjective cognitive impairment following COVID-19 infection that persisted for at least 12 weeks after the acute infection. The primary objective of this research was to investigate whether subjective cognitive impairment following COVID-19 infection can be objectified using neuropsychological assessments Further, we investigated whether psychiatric conditions, severity of the acute infection, peripheral inflammation markers, and the presence of *APOE*-ε4 predict objectively assessed cognitive impairment in these patients. For this purpose, a comprehensive neuropsychological assessment battery to evaluate attention, memory, and executive function was administered. Additionally, patients’ psychiatric symptoms were assessed using validated scales to capture the severity of depression and fatigue. Blood samples were gathered to analyze inflammation markers and *APOE* genotype.

## Methods

The present cross-sectional study was conducted from December 2021 to December 2023 at the Department of Psychiatry and Psychotherapy, Saarland University Hospital in Southwest Germany. A total of 229 patients sought treatment at our outpatient memory clinic for novel or exacerbated subjective cognitive impairments following a COVID-19 infection. Patients included in the study were at least 18 years old, gave their written informed consent prior to data collection, and provided a positive polymerase chain reaction (PCR) test as proof of a SARS-CoV-2 infection at least 12 weeks prior to data collection.

### Procedure and measurements

During the first appointment, patients completed questionnaires assessing depression, subjective fatigue severity, and alcohol use (~ 30 min). Subsequently, a clinical psychologist conducted a clinical interview focusing on obtaining subjective reports of cognitive disruptions in daily life, personal history of psychiatric conditions and corresponding treatments, as well as any signs of further psychopathological symptoms (~ 45 min). Thereafter, a computerized neuropsychological assessment was conducted, measuring aggravation of cognitive symptoms (~ 10 min), followed by the administration of a neuropsychological assessment battery to evaluate attention, memory, and executive functions (~ 60 min). Finally, blood samples were drawn. In two subsequent appointments, cranial magnetic resonance imaging (cMRI) and, if necessary, a lumbar puncture were carried out to rule out medical conditions that could explain cognitive dysfunction. These data were primarily used for diagnostic purposes and are therefore not systematically analyzed yet. Therefore, they are not specified in this study.

*History of psychiatric conditions* and *symptom severity of the acute COVID-19 infection* were based on patients’ reports.

*Depression* was measured using the German version of the Beck Depression Inventory 2 (BDI-II) [16], based on the DSM-IV criteria in the past two weeks.

*Subjective fatigue severity* was measured using the German version of the Fatigue Severity Scale [17], with mean scale scores ranging from 1 to 7. Mean values larger than 5 indicate severe fatigue.

*Cognitive symptom aggravation* was measured using a free recall (TUEGA; Test zur Überprüfung der Gedächtnisfähigkeit im Alltag) and a multiple choice recognition memory test (TUEGA-M), which are part of a computerized and validated neuropsychological forensic test battery [18]. Symptom aggravation is indicated when test takers recall < 5 out of 15 items in the free recall test or < 7 out of 15 in the multiple choice recognition test, or when they remember fewer items in the multiple choice recognition test compared to the free recall test.

*Objective cognitive performance* was measured using the Cognitive Basic Assessment Test set (COGBAT) of the Vienna Test System (VTS) [19]. This computerized test battery assesses three major cognitive domains with multiple tests per domain: attention (working speed, alertness, divided attention), memory (learning, short- and long-term free recall, recognition), and executive functions (working memory, planning, cognitive flexibility). Performance on each test is conveyed as a standardized score (T score), based on representative VTS norm tables.

Li-heparin monovettes were used by trained professionals to collect blood plasma, which were immediately sent to the central laboratory at Saarland University Hospital, where the plasma was examined for *peripheral inflammation markers* using the COBAS system. ECLIA was used to measure IL-6 and immunological turbidimetry was used to assess C-reactive protein (CRP). We determined the APOE genotype by restriction enzyme digestion of the appropriate PCR product according to a standard protocol. In brief, *APOE* PCR was performed using 100 ng of genomic DNA, rev-Primer 5′ GCC CCG GCC TGG TAC ACT GCC A 3′, forw-Primer 5′ TCC AAG GAG CTG CAG GCG GCG CA 3′ provided by metabion together with FailSafeTM PCR System (Epicentre). PCR product was digested by AflIII and Haell (New England Biolabs) and separated on a 3.5% native agarose gel for interpretation.

### Statistical analyses

Multivariate logistic and linear regression analyses were conducted to investigate the effects of several predictors on cognitive performance. Odds ratios and the incremental change in *R*^*2*^ (*R*^*2*^*Δ*) is reported as a measure of effect size for significant effects. Age, sex, years of formal education, actual medication for diabetes mellitus and hypertension, and time since the infection onset in months were included as covariates, as they might affect cognitive performance. History of psychiatric conditions was dichotomized (yes / no), as well as severity of the acute COVID-19 infection (without medical treatment/with medical treatment) and *APOE*-genotype (*APOE*-ε4 + / *APOE*-ε4-). Moreover, memory performance was calculated as the mean *T*-score between short- and long-term free recall and recognition. Learning performance was not considered in this study, as it is commonly confounded by attention and working memory abilities. Thus, the present memory performance score is a highly specific measure of episodic memory performance. *T*-scores < 40 (− 1sd) respective to representative norm tables were considered as objective cognitive impairment.

All statistical analyses were two-tailed with a significance level of α = 0.05, and were performed with RStudio 3.6.1 software (R Foundation for Statistical Computing, Vienna, Austria). For the sake of comparability, all continuous variables were *z*-standardized. The package “ggplot2” [20] was used to create the figures.

## Results

**Fig. 1:**
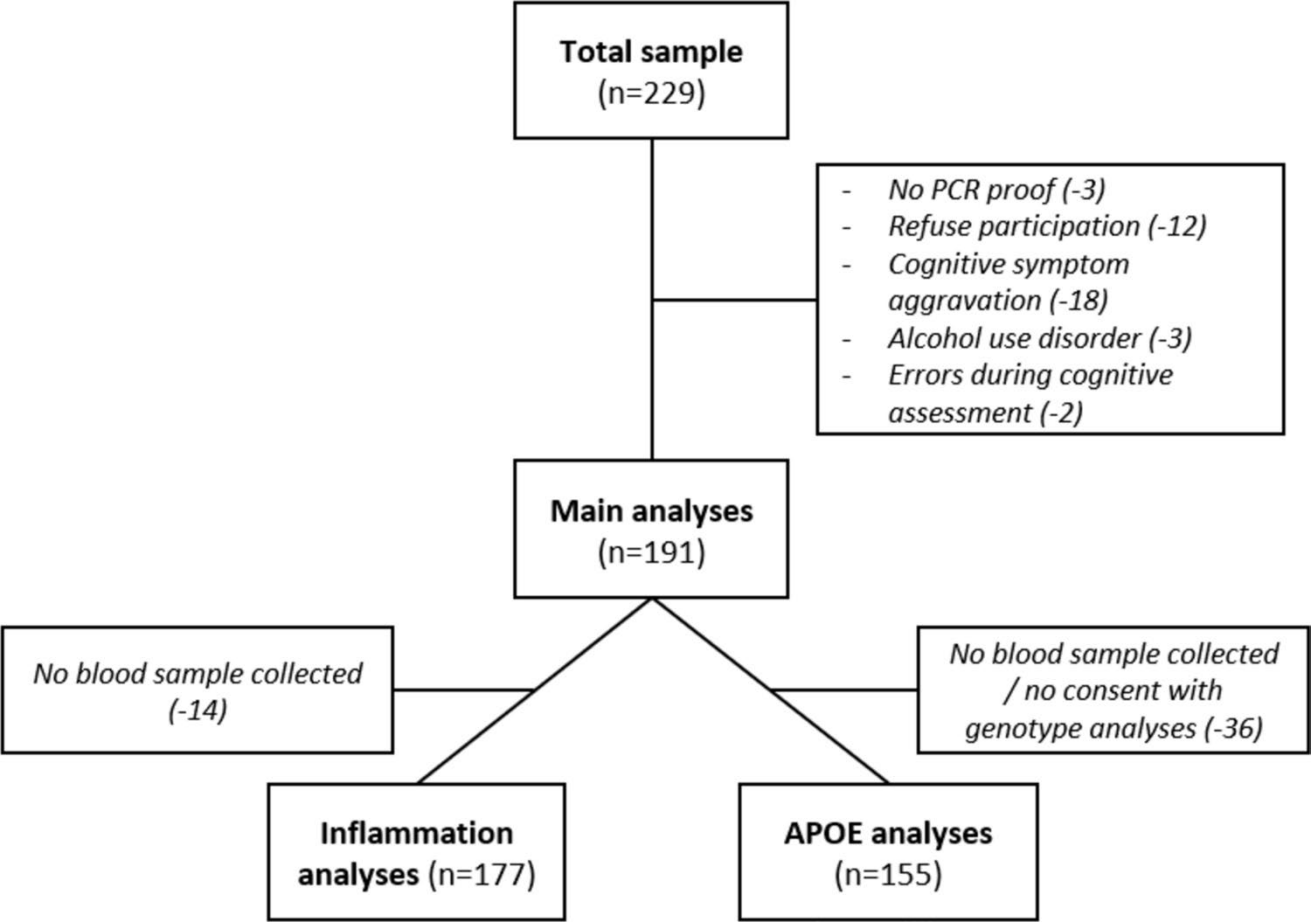
Exclusion flowchart. Note. For analyses including peripheral inflammatory markers the “Inflammation analyses” sample was used. For analyses including APOE genotype the “APOE analyses” sample was used. For all other analyses the “Main analyses” sample was used. *PCR* Polymerase-Chain-Reaction; *APOE* Apolipoprotein E

From the 229 patients that were investigated, data from 38 patients had to be excluded as they did not provide a positive PCR test proof (3), refused participation (12), showed signs of cognitive symptom aggravation (18), showed signs of alcohol use disorder (3) or because of technical errors during cognitive performance assessment (2). Thus, 191 (135 [70.68%] female) patients were included in the main analyses. Of the 191 patients in the main sample, we were unable to collect blood samples from 14 patients. Therefore, all analyses including peripheral inflammatory markers are limited to a sub-sample of 177 patients ("inflammatory analyses" sample). In addition, of the 191 patients in the main sample, the APOE genotype could only be extracted from 155 patients because blood samples were not collected or patients selectively refused genomic analyses. Therefore, all analyses including APOE genotype were restricted to a sub-sample of 155 patients (APOE analyses sample). The exclusion process is shown in Fig. 1.

**Fig. 2:**
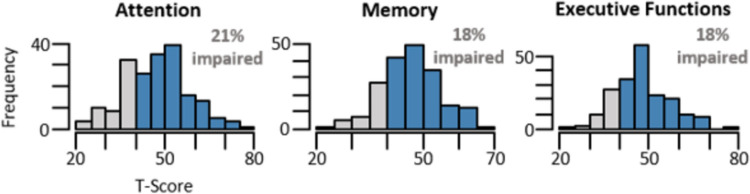
Histograms of T-scores of Cognitive Measures**.** Note. T-scores < 40 (objective cognitive impairment) are displayed in gray

Table 1 presents specific patient characteristics of our sample. On average, patients were evaluated about one year after the infection onset. During the acute COVID-19 infection, 79% of patients reported experiencing no symptoms or stayed in home quarantine with symptoms, while approximately 13% received medical treatment at home, and about 9% were hospitalized. About 46% of patients reported a history of psychiatric conditions, most frequently affective (~ 32%), as well as stress-related and somatoform disorders (~ 21%). Approximately 40% of patients reported currently being under psychiatric or psychotherapeutic treatment. BDI-2 scores indicated high rates of current depression in the study sample, with about 47% of patients meeting the criteria for moderate or severe depressive symptomatology. The majority of patients (79%) reported substantial subjective fatigue symptoms. Presence of the *APOE*-ε4 allele emerged in 31.6% (heterozygous) and in 0.06% (homozygous) of the patients. Regarding patients’ cognitive performance, the mean *T*-scores for attention, memory, and executive functions were 47.6 (*SD* = 10.85), 46.5 (*SD* = 7.89) and 47.9 (*SD* = 9.15), respectively. Objective cognitive impairment emerged in only 21% of patients for attention and 18% of patients for memory and executive functions each (see Fig. 2). In total, objective cognitive impairment in at least one of the domains was observed in 39% of patients.

**Table 1:** Patient characteristics for study population (n = 191)

Age	47.08 (19–79)
Female	135 (70.68%)
Formal education (years)	13.99 (8–21)
No graduation	1 (0.52%)
Lower secondary education	9 (4.71%)
Secondary / Vocational education	116 (60.73%)
High School	31 (16.23%)
Completed studies	34 (17.80%)
Employment	
Yes	155 (81.15%)
No	33 (17.28%)
Pension	3 (1.57%)
Months since infection	12.23 (2.57–39.7)
Vaccination status	
0	16 (8.38%)
1	7 (3.66%)
2	51 (26.70%)
3	112 (58.64%)
4	5 (2.62%)
Acute COVID-19 severity	
No symptoms	9 (4.71%)
Symptoms + home quarantine	141 (73.82%)
Medical care at home	24 (12.57%)
Hospitalization	15 (7.85%)
Mechanical ventilation	2 (1.05%)
History of psychiatric disorder (ICD-10)	87 (45.55%)
F10-19	2 (1.05%)
F30-39	61 (31.94%)
F40-48	40 (20.94%)
F50-59	3 (1.57%)
F90-98	5 (2.62%)
Actual psychic treatment	77 (40.31%)
Actual diabetes treatment	11 (5.76%)
Actual hypertension treatment	44 (23.04%)
Beck depression inventory	20.70 (3–53)
No depression	52 (27.23%)
Low depression	50 (26.18%)
Moderate depression	45 (23.56%)
Severe depression	44 (23.04%)
Fatigue severity scale	5.75 (1.55–7.00)
No fatigue	40 (20.94%)
Fatigue	151 (79.06%)
IL-6 (n = 177)	2.24 (1.5–12.3)
CRP (n = 177)	2.61 (0.6–28.6)
APOE-genotype (n = 155)	
22	2 (1.29%)
23	20 (12.90%)
24	4 (2.58%)
33	83 (53.55%)
34	45 (29.03%)
44	1 (0.06%)
Attention	47.61 (20–80)
Impaired	41 (21.47%)
Memory	46.48 (22.33–65.33)
Impaired	34 (17.80%)
Executive Functions	47.90 (20–80)
Impaired	35 (18.32%)

*Note.* Continuous variables are presented as mean (range) and categorical variables are presented as absolute values with percentages n (%). IL-6 and CRP (n = 177) and APOE-genotype (n = 155) are reported when collected. F10-19, Mental and behavioral disorders due to psychoactive substance use; F30-39, Affective disorders; F40-48, Neurotic, stress-related and somatoform disorders; F50-59, Behavioral syndromes associated with psychological disturbances and physical factors; F90-98, Behavioral and emotional disorders with onset usually occurring in childhood and adolescence; IL-6, Interleukin-6; CRP, C-reactive protein; *APOE*, apolipoprotein E

### Predictors of overall cognitive impairment

#### Covariates

Logistic Regression revealed that a one *SD* increase in age was significantly associated with a 1.66-fold elevated risk for cognitive impairment in any of the major cognitive domains (95%-CI = 1.21–2.32). Further, females were at 2.19-fold significant higher risk for cognitive impairment than males (95%-CI = 1.12–4.43), whereas one *SD* increase in years of formal education was significantly associated with a lower risk for overall cognitive impairment (OR = 0.65, 95% CI = 0.47–0.89). None of the other covariates (months since infection, actual diabetes and/or hypertension treatment) showed stronger associations.

#### Psychiatric conditions

**Fig. 3:**
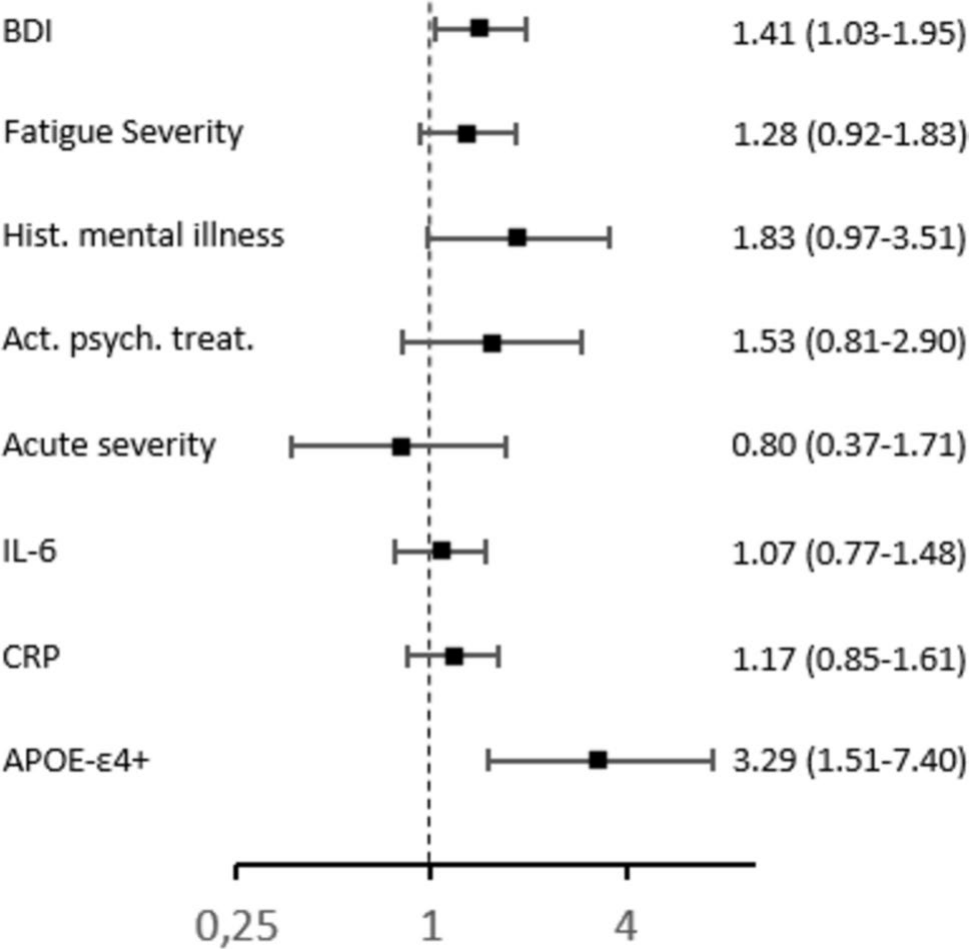
Predictors of Overall Cognitive Impairment. Note. Results of the logistic regression analyses. Impairment in any of the cognitive domains is coded as 0 = no impairment and 1 = impairment. Adjusted odds ratios and 95%-CIs are depicted on the right border. Adjusted odds ratios and CIs are controlled for age, sex, years of formal education, time since infection in months and current diabetes and hypertension medication. For visual display, the x-axis is logarithmically transformed. BDI, Beck depression inventory; IL-6, Interleukin 6; CRP, C-reactive protein; Hist., History of; psych., psychiatric/psychotherapeutic; *APOE*, Apolipoprotein E

Multivariate logistic regression revealed that a one *SD* increase in depressive symptomatology was strongly associated with a 1.41-fold elevated risk for impairment in any of the cognitive domains (95%-CI = 1.02–1.95), regardless of age, sex, years of formal education, time since infection, and medication for diabetes and hypertension. Moreover, *APOE*-ε4 carriers had a 3.29-fold higher risk for cognitive impairment (95%-CI = 1.51–7.40) as compared to non-carriers, independent from covariates. Fatigue severity, history of psychiatric conditions, current psychiatric or psychotherapeutic treatment, severity of the acute COVID-19 infection, and inflammation markers had no significant impact on cognitive impairment. Figure 3 depicts the results of the logistic regression analyses.

### Predictors of cognitive performance in single domains

#### Covariates

The impact of the covariates on cognitive domains is depicted in Table 2. Higher age and fewer years of formal education are significantly associated with decreased performance in attention, memory and executive functions. Females’ performance was significantly lower than males in attention and executive functions, and performance in memory and executive functions were significantly lower in patients who take diabetes medication. Time since infection and actual medication for hypertension had no impact on cognitive performance. Overall, the covariates explain 16% of the variance in attention performance, 14% of the variance in memory performance and 18% of the variance in executive functions performance.

**Table 2:** Univariate impact of covariates on cognitive performance

	Attention			Memory			Executive Functions		
	*Beta*	*p*	*R*^*2*^	*Beta*	*p*	*R*^*2*^	*Beta*	*p*	*R*^*2*^
Age	– 0.28	< 0.001	0.08	– 0.18	0.013	0.03	– 0.23	0.001	0.05
Sex	– 0.43	0.006	0.04	– 0.09	0.578	< 0.01	– 0.39	0.015	0.03
Formal education (years)	0.20	0.007	0.04	0.30	< 0.001	0.09	0.28	< 0.001	0.08
Months since infection	– 0.09	0.197	0.01	– 0.03	.713	< 0.01	– 0.10	0.179	< 0.01
Actual diabetes treatment	– 0.19	0.536	< 0.01	– 0.87	0.005	0.04	– 0.84	0.006	0.04
Actual hypertension treatment	– 0.05	0.744	< 0.01	– 0.26	.130	0.01	– 0.24	0.167	0.01
Total			0.16			0.14			0.18

*Note.* Total R^2^ refers to the multivariate model including all covariates as predictors

#### Psychiatric conditions

**Fig. 4:**
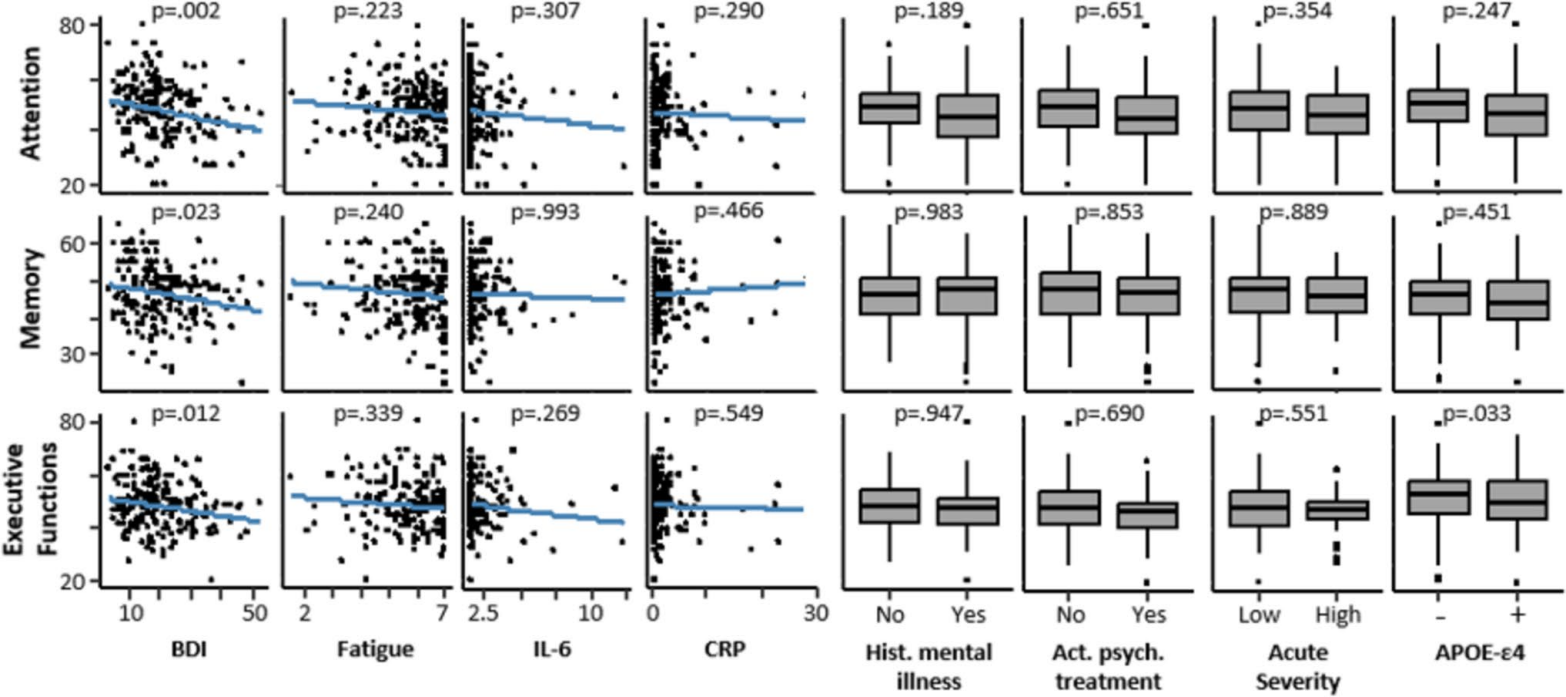
Predictors of Cognitive Performance. Note. Blue charts in scatterplots show the linear trend. Lower and upper hinges of the boxplots correspond to the first and third quartiles. Medians are displayed within boxplots. Whiskers extend until ± 1.5*IQR. Single data points beyond are considered as outliers. p-values refer to the analyses described in the results section and are controlled for age, sex, years of formal education, time since infection in months and current diabetes and hypertension medication. BDI, Beck depression inventory; IL-6, Interleukin 6; CRP, C-reactive protein; Hist., History of; psych., psychiatric/psychotherapeutic; APOE, Apolipoprotein

BDI-2 scores predicted the performance in attention (*β* = − 0.21, *t*(183) =  − 3.14, *p* = 0.002, *R*^*2*^*Δ* = 0.04), memory (*β* = − 0.16, *t*(183) =  − 2.30, *p* = 0.023, *R*^*2*^*Δ* = 0.02) and executive functions (*β* = −0.17, *t*(183) =  − 2.53, *p* = 0.012, *R*^*2*^*Δ* = 0.03), independent of age, sex, years of formal education, time since infection, and medication for diabetes and hypertension. Patients with higher BDI-2 scores, indicating more severe depressive symptomatology, had significantly lower performance in all three domains measured with the COGBAT. *APOE*-ε4 carriers showed significantly worse performance in executive functions (*β* = − 0.35, *t*(183) = −2.15, *p* = 0.033, *R*^*2*^*Δ* = 0.03), but not in attention (*p* = 0.247) or memory (*p* = 0.451), independent of covariates. Fatigue severity did not predict cognitive performance in any of the analyses. Neither the history of psychiatric conditions, nor current psychiatric or psychotherapeutic treatment predicted the performance in any of the cognitive domains. Regarding acute COVID-19 infection severity and inflammation markers at the time of testing, no significant associations were found for attention (*p*-values = 0.29–0.354), memory (*p*-values = 0.466–0.993) and executive functions (*p*-values = 0.269–−0.549). Analyses are shown in scatterplots and boxplots in Fig. 4.

## Summary

Taken together, age and fewer years of formal education were positively and negatively associated with cognitive impairment. Females showed higher risk for cognitive impairment, but only in attention and executive functions. While taking diabetes medication was not associated with cognitive impairment overall, it had adverse effects on performance in memory and executive functions.

Depressive symptoms and *APOE*-ε4 burden were the primary predictors of cognitive impairment. Overall, depressive symptoms were consistently associated with declining performance in all cognitive domains, whereas *APOE*-ε4 was only associated with worse performance in executive functions.

## Discussion

Novel or worsening subjective cognitive impairment and depressive symptoms are frequently reported by Post-COVID patients. The present study investigated whether such complaints can be objectified using neuropsychological testing and examined how impairment manifested across cognitive domains in patients who voluntarily sought help in our outpatient memory-clinic. Our findings revealed that approximately 39% of the patients had objective impairment in at least one of the domains. Higher rates of depression significantly increased the risk of objective impairment across all cognitive domains. Additionally, the presence of the *APOE*-ε4 allele was associated with an increased risk of objective cognitive impairment, particularly in executive functions. Fatigue severity, the acute severity of COVID-19, and peripheral inflammation markers (IL-6, CRP) at time of testing did not show any impact on cognition.

With 71% female participants, a mean age of 47 years, and substantial subjective fatigue in 79% of the cases, the present study sample aligns with major epidemiological findings, which identify female sex and middle to higher age as risk factors for developing Post-COVID conditions [2, 21]. Almost one half of our patients (46%) reported a history of psychiatric diseases, particularly affective, and stress-related or somatoform disorders. This exceeds the average prevalence rates of depressive and anxiety disorders for the European region [22] and is even higher than the prevalence rates of depression during the COVID-19 outbreak [23]. Moreover, about 47% showed features of moderate to severe depressive symptoms. These rates seem plausible, as a large prospective cohort study showed that pre-infection psychological distress, especially depression and anxiety, is a risk factor for developing Post-COVID conditions following COVID-19 [24]. However, these findings suggest that our study sample is particularly psychologically distressed.

### Divergence between subjective and objective cognitive impairment

Consistent with a previous meta-analysis [5], our major finding indicates that about one-fifth of patients are objectively impaired in each cognitive domain. However, when considering all cognitive domains collectively, approximately 39% of the patients exhibited objective cognitive impairment. Many earlier studies relied on subjective reports or screening procedures, which may underestimate the frequency of impairment in specific cognitive domains. This highlights the strength of the present study, which employed a standardized, computerized test battery. On the other side, it is crucial to mention, that despite suffering from severe subjective cognitive complaints (inclusion criteria), more than half of our patients did not exhibit objective impairments as measured by standardized tests.

Accordingly, a study on patients that survived intensive care treatment for severe COVID-19 found no correlation between subjective cognitive and objective cognitive impairment six months after hospital discharge [25]. Previous research showed that subjective cognitive impairment is more likely associated with depression rather than with objective impairment [26–28]. As such, depression can negatively bias self-evaluation and self-esteem, affecting subjective appraisal of cognitive functioning even when objective performance is unimpaired [29]. This aligns with the high frequency and intensity of depressive symptoms observed in our study. Another explanation for the divergence between subjective and objective cognitive impairment in our patients may be that patients maintain normal cognitive performance through compensatory neural processing and the activation of alternate brain regions [30].

These findings have some important clinical implications for the evaluation and management of subjective cognitive impairment following infection with COVID-19. Subjective cognitive impairment can be economically assessed. However, its clinical validity is still debated [31], especially in less educated populations and men [32, 33]. It is also noteworthy that subjective cognitive complaints have higher predictive power for objective impairment when they are aligned with distinct cognitive processes and target valid examples of cognitive problems in daily life [34, 35]. Unfortunately, subjective complaints after COVID-19 infection are often inaccurately referred to as "brain fog" or a "fuzzy brain state" [6]. Furthermore, objective cognitive impairment after COVID-19 infection has a high inter-individual variance (e.g., memory, language, attention, multimodal perception, and executive functions) and is often accompanied by depression, fatigue, or sleep disturbances [6]. Therefore, and in light of our findings, we recommend comprehensive neuropsychological testing and assessment of psychiatric conditions in post-COVID-19 patients with subjective cognitive impairment.

### Determinants of objective cognitive impairment

In the present study, depression was consistently associated with cognitive impairment across all cognitive domains. This link is well-established in the literature [36, 37]. It has been hypothesized that in the post-COVID state, there may be a prolonged systemic inflammatory state that predisposes patients to persistent depression and associated neurocognitive dysfunction [10]. This hypothesis is based on previous work proposing several pathways by which peripheral inflammation and cognitive impairment may be linked. For example, persistent peripheral inflammation could lead to disruption of the blood–brain barrier, resulting in neuroinflammation that could lead to neurodegeneration and adverse cognitive effects [38]. It has been repeatedly shown that peripheral markers of inflammation such as IL-6 and CRP are associated with cognitive impairment as a function of aging [39]. However, we did not observe an association between cognitive function and peripheral inflammatory markers or the acute severity of COVID-19. This finding is consistent with a recent study showing no association between a broad range of peripheral inflammatory markers and cognitive impairment in post-COVID patients over 18 months [40]. As cognitive impairment in this study was based on subjective reports of initially hospitalized patients, the result is well complemented by our findings in patients who underwent extensive neuropsychological assessment. However, another study showed that serum levels of interleukin-10, interferon-gamma, and sTREM2 are elevated in post-COVID patients, especially in those with cognitive impairment [41]. These results may differ from our study because they include the assessment of different peripheral inflammatory markers and the patients in that study had no history of mental or neurological disorders, nor any psychiatric history in their first-degree relatives. In contrast, almost half of the patients in our study reported a psychiatric history and/or moderate to severe depressive symptoms. This highlights the importance of assessing psychiatric conditions in post-COVID patients in research and clinical practice.

Additionally, our findings indicate no association between cognitive impairment and subjective fatigue. Recently, Pan et al. (2019) [42], suggest a bidirectional link: cognitive impairment in depression hinders psychosocial functioning, which in turn elevates and maintains depressive symptoms. Thus, addressing cognitive impairment could alleviate depressive symptoms and vice versa. As a consequence, several potential therapeutic strategies addressing both comorbidities have been suggested including cognitive remediation, cognitive behavioral therapy and treatment with conventional antidepressants. Two recent studies have demonstrated substantial improvements in Post-COVID patients treated with selective serotonin reuptake inhibitors (SSRIs). Mazza et al. (2022) [43], demonstrated a rapid reduction of depressive symptoms in a Post-COVID depression population, while Rus et al. (2023) [44], reported substantial improvements in “brain fog” and sensory overload in a Post-COVID population without clinical depression. Additionally, there is evidence suggesting a gradual reduction in fatigue, somatic symptoms, and concentration problems, alongside improved physical and social functioning over a one-year period in a Post-COVID cohort treated with cognitive behavioral therapy [45].

Our study showed that *APOE*-ε4 carriers are at an elevated risk for cognitive impairment, particularly in executive functions. To our knowledge, this is the first study that explores this link in a Post-COVID population, using extensive standardized neuropsychological testing. Our results thus complement previous, varying findings from studies that in part relied solely on screening tools or subjective symptom reports [13–15]. The association between *APOE*-ε4 and cognitive impairment is not unique to Post-COVID conditions. A meta-analysis [46] found that non-demented healthy-aging *APOE*-ε4 carriers exhibited reduced global cognitive performance, episodic memory and executive functions compared to non-carriers. Additionally, a longitudinal study [47] has shown that *APOE*-ε4 allele carriage is associated with lower IQ-scores during childhood and adolescence, especially in females, which potentially contributes to the development of cognitive impairment later in life. This may further explain why females more often experience cognitive impairment following COVID-19. *APOE*-ε4 contributes to cognitive decline and Alzheimer’s disease through various mechanisms, including amyloid-beta deposition, tau-mediated neurodegeneration, neuroinflammatory responses, lower cholesterol transport capacity and increased lipid droplet accumulation, vascular dysfunction, and endothelial blood–brain-barrier dysfunction [48, 49]. These mechanisms represent several possible therapeutic targets for addressing cognitive decline after COVID-19 infection.

### Limitations and future directions

Our study has several limitations. Firstly, the cross-sectional design inherently restricts causal interferences. The study sample is selectively biased, as individuals with prior contact with psychiatric health-care might be more inclined to seek psychiatric help, which could explain the high rates of mental health history and acute depression in our sample. Therefore, our results may not be generalizable to the broader population of Post-COVID patients, especially those primarily experiencing somatic symptoms. Moreover, the cross-sectional design limits statistical variance for peripheral inflammation markers, potentially hampering analyses. The study was also not designed to compare patients with mild versus severe disease-courses of acute COVID-19, necessitating cautious interpretation of the results and validation in specifically designed studies. Secondly, we did not have access to pre-infection cognitive function data for our patients. It remains unclear if cognitive impairment existed prior to the acute COVID-19 infection or if it is directly or indirectly associated with COVID-19. Additionally, due to the patient recruitment design, we lacked a control group without subjective cognitive impairment after COVID-19, which decreases the specificity of the observed deficits.

Future research should focus on specific Post-COVID subgroups to derive individualized treatment options. Controlled studies are needed to compare patients with varying inflammation states during the acute and post-acute phase of the disease. This is crucial for understanding the associations between disease severity, inflammation status, depression, and cognitive impairment in Post-COVID. Lastly, longitudinal data are warranted to disentangle the bidirectional link between depression and cognitive impairment in Post-COVID patients.

## Conclusion

In this cross-sectional study, we found that subjective cognitive impairment following COVID-19 was not objectifiable in more than half of the examined patients, suggesting that these impairments may be more closely related to depressive symptoms rather than actual cognitive deficits. The prevalence of depressive symptoms was notably high in our sample of Post-COVID patients and was associated with objective cognitive impairment across different cognitive domains. Given that effective treatments for depression can also improve cognitive impairment, it is imperative to conduct extensive neuropsychological testing and assess depressive symptoms in Post-COVID patients, especially those reporting subjective cognitive decline. Furthermore, our data reveal a link between objective cognitive impairment and *APOE*-ε4 burden, which may not be exclusive to the Post-COVID condition. Future studies should evaluate *APOE*-ε4-mediated neurodegenerative mechanisms as potential therapeutic targets for cognitive impairment following COVID-19.
